# Understanding the Colloidal and Hydration Control in Rheological Evolution of 3D Printed MgO-SiO_2_-K_2_HPO_4_ Gel System

**DOI:** 10.3390/gels11100827

**Published:** 2025-10-14

**Authors:** Xianhuan Cai, Fan Chen, Zhihui Zhao, Peng Xiao, Yujuan Zhang

**Affiliations:** College of Civil Science and Engineering, Yangzhou University, Yangzhou 225127, China

**Keywords:** magnesium potassium phosphate cement, MgO-SiO_2_-K_2_HPO_4_, colloidal-hydration synergy, rheological property, 3D printing

## Abstract

Monitoring the time-dependent rheological properties of 3D printed MgO-SiO_2_-K_2_HPO_4_ is critical for optimizing the dynamic structural reconstruction ability. The collaborative analysis for the contribution of colloidal force based on EDLVO theory and the volume fraction of K-struvite (MgKPO_4_·6H_2_O) was conducted. Results showed that 20% silica fume (SF) was identified as the optimal content to achieve balanced rheo-mechanical performance (28 d compressive strength = 113.63 MPa, dynamic yield stress = 359.98 Pa, thixotropic area = 2.14 × 10^4^ Pa/s). The static yield stress development within 50 min exhibited two distinct stages: the initial rapid linear growth stage (Stage I, 5–30 min) dominated by colloidal forces (R^2^ = 0.81 at 20% SF), followed by the slow increased plateau (Stage II, 30–50 min) correlated with K-struvite volume fraction. Also, dual crystallization pathways of K-struvite included direct precipitation from supersaturated Mg^2+^, K^+^, PO_4_^3−^ ionic species and transformation from potassium-deficient phosphate phase. Quantitative results establish a predictive framework for microstructural construction, enabling precise control of structural build-up and 3D printability in MgO-SiO_2_-K_2_HPO_4_ cementitious composites.

## 1. Introduction

3D printed concrete (3DPC), as a semi-automated and intelligent technology, has attracted increasing attention in construction engineering [1,2]. Materials eligible for 3D printing should possess favorable rheological properties, requiring reduced dynamic yield stresses to enhance pumpability from the nozzles [3], increased static yield stresses to reduce deformation for improved buildability [4], and high thixotropy to ensure smooth extrusion flow during shearing and maintain sufficient plastic strength at rest [5].

Magnesium potassium phosphate cement composites (MKPCs) are typically produced through the rapid acid–base reaction between dead burnt magnesia (MgO) and soluble potassium dihydrogen phosphate (PDP, KH_2_PO_4_) [6,7]. We have found that the superior early strength and controlled rheological properties of MKPCs demonstrate the potential for 3D printing [8]. Nevertheless, traditional MKPCs prepared with MgO-KH_2_PO_4_-retarder exhibit rapid setting and high heat release, which increase the risks of extrusion failure through nozzle clogging [9]. Moreover, incorporating retarders diminishes the early strength of traditional MKPCs of MgO-KH_2_PO_4_-retarder due to the delayed K-struvite nucleation. While potassium monohydrogen phosphate (K_2_HPO_4_·3H_2_O) serves as an alternative phosphate for MKPCs, prior studies revealed that fully replacing KH_2_PO_4_ with K_2_HPO_4_·3H_2_O resulted in limited strength development (<15 MPa at 1d) [10]. However, we now report that silica fume (SF) can function as the reactive chemical bridge, enabling the MKPCs prepared with MgO-SiO_2_-K_2_HPO_4_ with superior strength and tailored rheological properties for extrusion-based 3D printing [11]. This strategy achieves simultaneous enhancement of early thixotropic area (5.94 × 10^4^ Pa/s, +22.73% vs. control group) and ultimate 28 d compressive strength (133.89 MPa, +557.61% vs. control group), governed by the SF-driven reaction involving MgO-K_2_HPO_4_·3H_2_O. The active SiO_2_ in SF can promote the release of Mg^2+^ formed by hydrolysis of dead-burnt MgO and accelerate K-struvite phase crystallization [12]. Moreover, the leaching of Ca^2+^, Al^3+^ and Si^4+^ from slag powder into the high pH aqueous of the MgO-SiO_2_-K_2_HPO_4_ system drives the formation of C-(A)-S-H gel in later hydration, which further enhances the mechanical strength and water resistance of the novel MKPCs [13].

Static yield stress, defined as the critical stress required to maintain structural stabilization by flocculation and hydration-derived chemical bonds in resting cement pastes [14,15], is principally governed by two competing mechanisms: interparticle colloidal forces and hydration-induced network strengthening [16]. Upon contact with water, MKPCs undergo rapid dissolution, and generate reversible colloidal forces within a few seconds [17]. These transient colloids directly govern the plastic structural strength of the wet matrix by modulating interparticle spacing and stress transmission [18,19,20]. Colloidal interactions, which primarily include van der Waals forces, electrical double layer forces and acid–base interaction forces, are quantifiable by Extended DLVO (EDLVO) theory [21,22], which provides a framework for predicting floc stability. Unlike the Portland cement system where structural evolution is dominated by the volume fraction of C-S-H gel, early-stage hydration of the MgO-SiO_2_-K_2_HPO_4_ system involves the competitive formation of M-S-H gel, C-(A)-S-H gel, brucite (Mg(OH)_2_) and K-struvite (MgKPO_4_·6H_2_O) [23]. While brucite inhibits K-struvite crystallization by competing Mg^2+^ ions [24], the nucleation rate of K-struvite directly affects the static yield stress [16]. Zhao et al. [25] demonstrated that increasing the MgO/phosphate (M/P) mass ratio with borax additives further enhanced the buildability of MKPCs by retardation-controlled hydration. Conversely, Li et al. [26] reported that partial MgO substitution with 20% fly ash reduced the static yield stress by 31.89 Pa but improved the thixotropy, highlighting the balance requirement between printability and structural recovery. Critically, existing studies predominantly focus on the compositional optimization (e.g., admixtures, M/P ratios) to meet empirical rheological targets, neglecting mechanistic linkages between hydration, flocculation and time-dependent evolution of static yield stress.

This study focuses on the systematic assessment of the MgO-SiO_2_-K_2_HPO_4_ system as 3D-printable cementitious material by quantifying its rheological properties, including dynamic yield stress (τ_0_), thixotropic area (A), static yield stress (τ_s_), plastic viscosity (μ) and structural parameter. The effects of SF content are studied. The influences of particle physical properties, electrodynamics and hydration phase evolution on time-dependent static yield stress enhancement are systematically investigated. Crucially, the particle level framework is established by linking yield stress evolution to colloidal forces by EDLVO theory quantification and K-struvite volume fraction by XRD-Rietveld quantification. Preliminary evidence suggests that inclusion of SF can contribute to the understanding of printability–stability optimization through the nucleation site.

## 2. Results and Discussion

### 2.1. Setting Time Results

**Figure 1:**
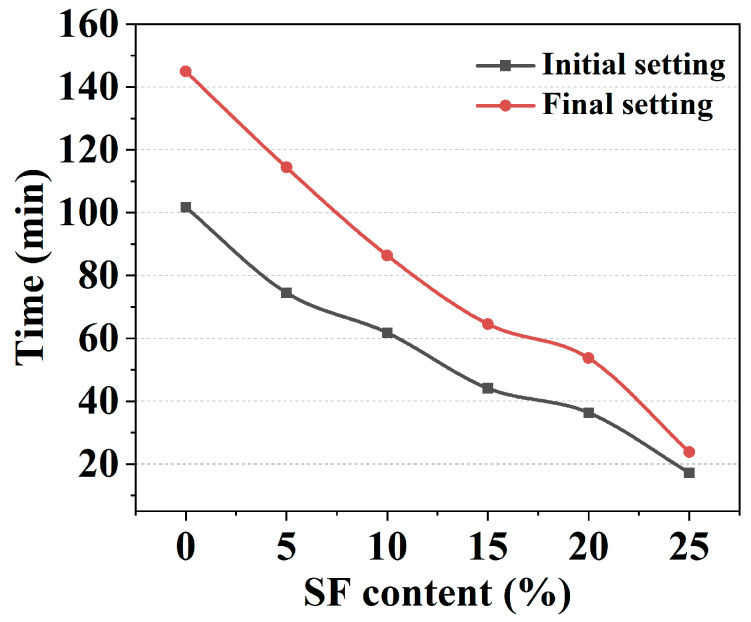
Initial and final setting time of mixtures with varying SF content.

Figure 1 demonstrates the effect of SF content on slurry setting behavior. As SF content increased from 0% to 25%, both initial and final setting times decreased substantially from 101.38 min to 17.24 min and 144.93 min to 23.68 min, respectively. This acceleration primarily resulted from the accelerated dissolution of Mg(OH)_2_ by consuming more OH^−^ ions and the highly specific surface area of SF increasing water demand [11,12]. Notably, at 25% SF content, the setting time fell significantly below the operational window required for 3D printing, markedly elevating the extrusion nozzle-clogging risk due to premature rheological property evolution. Consequently, practical printability should restrict SF content within 20% to ensure that the slurry maintains adequate liquidity throughout the deposition process. In the subsequent research, the SF content above 20% was not considered.

### 2.2. Rheological Property Results

**Figure 2:**
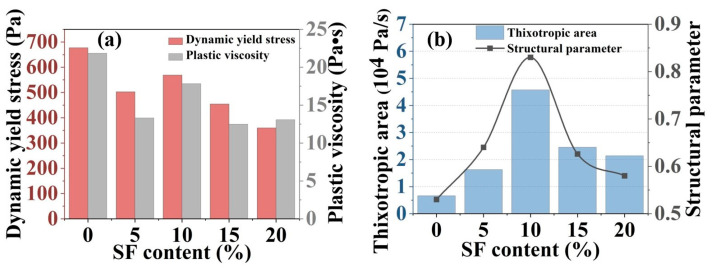
(**a**) Dynamic yield stress and plastic viscosity and (**b**) thixotropic area and structural parameters with varying SF contents.

The dynamic yield stress and plastic viscosity results were derived from the Bingham model fitting with the shear rate range of 10–90 s^−1^, as shown in Figure 2a. Increasing SF contents from 0% to 20% induced a reduction in dynamic yield stress and plastic viscosity, with values declining from 677.11 Pa to 359.98 Pa and from 21.84 Pa·s to 13.08 Pa·s, respectively. This trend was primarily attributed to the characteristic spherical geometry and low surface asperity of SF particles, which minimized the interparticle friction [27].

Thixotropic behavior was quantified by thixotropic area (A) and structural parameters, respectively, revealing the nonlinear dependence on SF content, as depicted in Figure 2b. The thixotropic area of the mixtures peaked at 4.58 × 10^4^ Pa/s with 10% SF content, then declined to 2.14 × 10^4^ Pa/s at 20% SF, while the structural parameters exhibited a similar trend and increased to 0.83 at 10% SF before declining at higher SF contents. Optimal SF incorporation enhanced thixotropy through improved particle packing density and accelerated formation of M-S-H gel by SF-MgO surface reactions [12]. Conversely, as the content of SF exceeded 10%, excessive SF triggered localized agglomeration, disrupting slurry homogeneity and generating shear-induced stress concentrations that destabilized thixotropic recovery.

**Figure 3:**
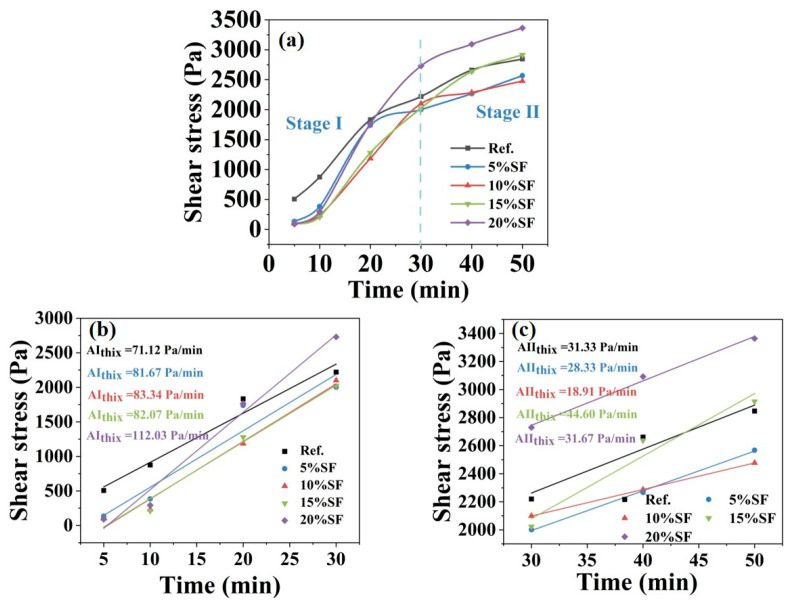
(**a**) Time evolution of static yield stress with varying SF contents, and the rate of growth of (**b**) Stage I and (**c**) Stage II.

The time evolution of static yield stress in the MgO-SiO_2_-K_2_HPO_4_ system was systematically investigated with varying SF contents, as illustrated in Figure 3. A biphasic growth pattern in the static yield stress based on the growth rate (A_thix_) emerged: Stage I (0–30 min) exhibited rapid linear growth, with A_thix_ increasing significantly from 71.12 of the control group to 112.03 Pa/min of the mixture with 20% SF; Stage II (30–50 min) exhibited a decelerated trend, with A_thix_ of the mixture with 20% SF dropping to 31.67 Pa/min. Notably, although the mixture with 20% SF initially displayed a lower static yield stress than the control group within the first 20 min, the stress surpassed the control group after 20 min, reaching a peak value of 2729.84 Pa at the end of Stage I, which was critical for dimensional stability in 3D printing. This behavior originated from SF-driven enhanced colloidal network restructuring and precipitation of K-struvite, which synergistically balanced early workability before 20 min and rapid structural build-up after 20 min.

### 2.3. Mechanical Property and 3D Printing Results

**Figure 4:**
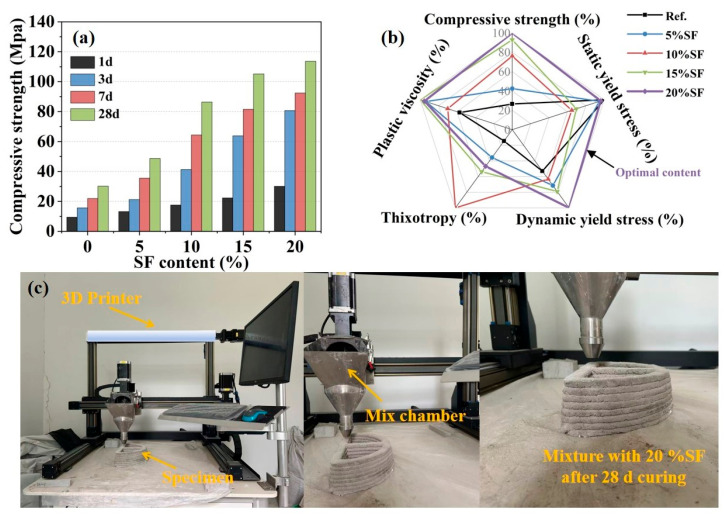
(**a**) Compressive strength of mixtures with varying SF contents, (**b**) radar map of various properties and (**c**) 3D printer and 3D printed specimen.

The compressive strengths measured at different ages are presented in Figure 4a. Specimens with 20% SF (30.14 Mpa) exhibited a 218.27% increase in 1 d compressive strength compared to the control group (9.47 Mpa). Furthermore, the 28 d compressive strength attained 113.63 MPa when the SF content reached 20%. The incorporation of SF significantly enhanced the mechanical properties and still developed greatly over time, as shown in a previous study [11]. K-struvite plays the dominant role in mechanical properties and is still the main hydration product of the MgO-SiO_2_-K_2_HPO_4_ system. The addition of SF increased production and crystallinity of K-struvite, which was conducive to pore structure optimization and enhanced the mechanical strength of the hardened pastes.

A multivariate radar plot quantified trade-offs among rheological parameters (dynamic yield stress, plastic viscosity, thixotropy and static yield stress) and compressive strength, as shown in Figure 4b. Although the control group exhibited the highest static yield stress, the significantly elevated plastic viscosity and dynamic yield stress increased the risk of blockage or uneven extrusion. When the content of SF was less than 10%, reduced dynamic yield stress and plastic viscosity alleviated the fluidity problem, but limited compressive strength development, which made it difficult to meet the long-term service requirements. When the content of SF was greater than 10%, compressive strength optimization was achieved at the expense of static yield stress reduction, but raised structural collapse risk. It can be worth noting that the mixture with 20% SF demonstrated balanced performance. The moderate increase in the thixotropy ensured the instantaneous structural self-support capacity after extrusion. The relatively high static yield stress maintained the stability of the printed filament, while the compressive strength reached the optimal value of 113.63 MPa. Although its thixotropy was lower than that of the 10% SF mixture, the process of adaptability compensation of the rheological properties can be achieved by adjusting the extrusion pressure (e.g., 0.1 Mpa) and printing speed (e.g., 10 mm/s). As shown in Figure 4c, the 3D printing process was verified using the mixture optimized by 20% SF. During the continuous printing process, the slurry demonstrated excellent rheological adaptability and structural stability, successfully completing the layer-by-layer deposition of the entire component within 10 min. After 28 d curing, the printed specimen still maintained precise geometric profiles and dimensional stability. It was confirmed that 20% SF content was the optimal content for rheological–mechanical co-optimization, providing a quantitative proportion–optimization strategy for the design of 3D printed MgO-SiO_2_-K_2_HPO_4_.

### 2.4. Particle Physical Properties Results

**Figure 5:**
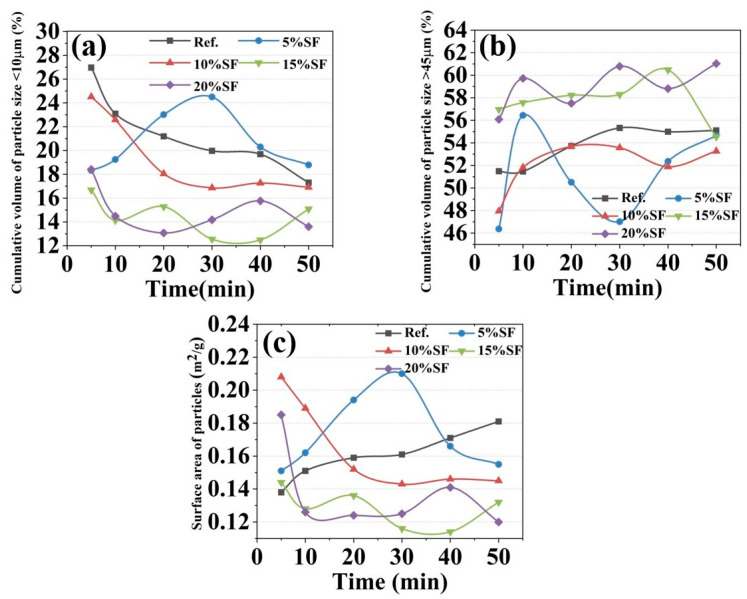
The variation in (**a**,**b**) particle size distribution, (**c**) surface area with hydration age and SF contents of 3D printed MgO-SiO_2_-K_2_HPO_4_.

Fresh 3D printed MgO-SiO_2_-K_2_HPO_4_ system pastes are complex multiphase systems comprising water, solid particles and porosity. Rheological behavior is influenced by particle characteristics including size distribution, morphology and specific surface area [28,29]. Figure 5a,b show the influence of hydration age on the cumulative volume of particle sizes < 10 μm and >45 μm, respectively. The cumulative volume of particles < 10 μm decreased with hydration age, whereas those of >45 μm increased, indicating SF-participated hydration producing larger microscopic particles [30,31]. It should be particularly pointed out that the particle size of colloidal particles measured within 50 min was different from the mesoscopic pore structure of the 14 d hardened mixtures by mercury intrusion porosimeter in previous studies [11]. The changes in specific surface area of particles with hydration ages for varying SF contents are shown in Figure 4c. The specific surface area generally declined with the increasing SF content, which is likely be attributed to the formation of highly viscous layers on the particle surface by SF dissolution enhancing interparticle adhesion and agglomeration [32]. Furthermore, reduced specific surface area diminished the adsorbed water film thickness and increased the interparticle space, thereby decreasing the electrical double layer forces (F_LW_) between particles and improving the pumpability [33,34].

**Figure 6:**
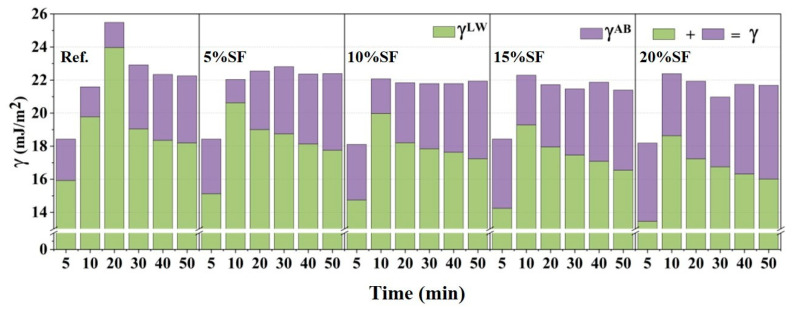
The surface energy (γ), apolar surface energy (γ^LW^) and polar surface energy (γ^AB^) of particles of 3D printed MgO-SiO_2_-K_2_HPO_4_.

The particle surface energy (γ) governs interparticle interactions during hydration through the polar (γ^AB^) and apolar (γ^LW^) components. Here, the γ^AB^ denotes the intermolecular interaction between Lewis acids (electron acceptors) and bases (electron donors) on a solid surface, where increased γ^AB^ correlates with enhanced surface hydrophilicity. The γ^LW^ denotes the Lifshitz–van der Waals interaction, mediating electrostatic attraction through apolar and intermolecular forces [35]. The variation in γ with hydration age is depicted in Figure 6. Hydration induced similar evolution in γ^LW^ and γ, peaking at intermediate ages before declining, whereas γ^AB^ increased monotonically. These trends were related to surface and structural reorganization due to the early hydration reaction. The incorporation of SF reduced the γ^LW^ and γ and accelerated peak emergence, which is primarily attributed to SF acting as a nucleation site for M-S-H gel, which introduced abundant silanol (Si-OH) groups on particle surfaces and modified surface interfacial energy equilibria [36]. Contrary to a prior report [20], the γ^LW^/γ^AB^ ratio and static yield stress showed no significant correlation with static yield stress, yet surface energy directly influenced interparticle forces, thereby modulating the static yield stress.

### 2.5. Electrodynamic Parameter Results

**Figure 7:**
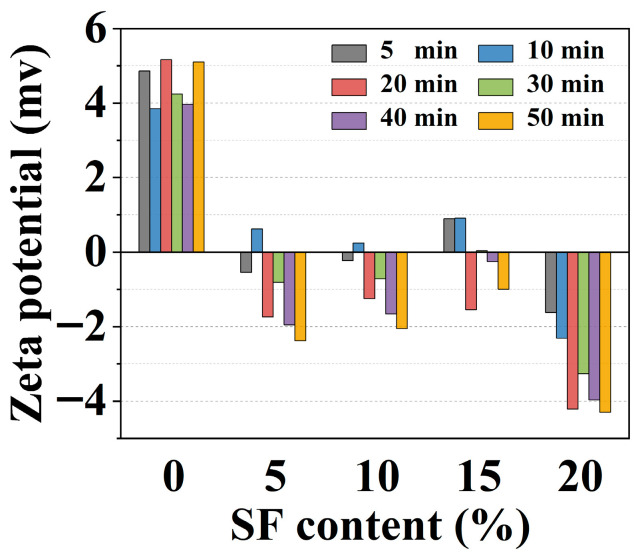
The changes in zeta potential (ξ) of 3D printed MgO-SiO_2_-K_2_HPO_4_ with hydration age and SF contents.

The zeta potential (ξ), quantifying the electrokinetic potential at the particle–liquid interface, reflects the surface charge characteristics of suspended particles in electrolyte solutions. As shown in Figure 7, the ξ of the control group remained relatively stable within the range of 3.85–5.16 mV. The absolute value of ξ increased progressively with hydration age, while SF incorporation induced the negative ξ (e.g., ξ = −4.29 mV with 20% SF at 50 min). This charge reversal stemmed from SF-driven nucleation of M-S-H gel. The surface of M-S-H exposed many hydroxyl groups (-OH), which can be dissociated in water. Following H^+^ release, the residual [Si-O^−^] groups on the surface make the M-S-H gel negatively charged [37]. The fluidity and compatibility of cement paste were influenced by particle dispersion and the development of surface charge affected the dispersion effect [38]. It was reported that the electric double layer force was proportional to ξ^2^ [39], indicating that the control group with higher absolute values of ξ tended to have superior double layer forces, reducing net interparticle attraction (i.e., lower cohesion). Increased SF adsorption on the particle surface led to greater repulsive forces between the particles, enhancing the fluidity and pumpability of the mixtures.

In colloidal suspensions, counterions are adsorbed onto particle surfaces to form a Stern layer. The ξ, measured at the slipping plane which is the boundary between the Stern layer and the diffuse layer, approximates the Stern potential in colloidal systems [40]. Ion adsorption predominantly occurs within the Stern layer, thereby altering the ion hydration radii. The incorporation of SF enhanced Mg^2+^ and K^+^ counterions adsorption on negatively charged surfaces. The high ionic concentration in the Stern layer induced partial desorption of counterions from hydration shells, reducing their effective hydration radii. This facilitated ion transport across the Stern layer to the surface, improving hydration kinetics through improved ion accessibility and reactive sites. This demonstrated the positive role of SF in adjusting colloid interactions and promoting the generation of hydration products, which improved the establishment of structural networks for 3D printing.

**Figure 8:**
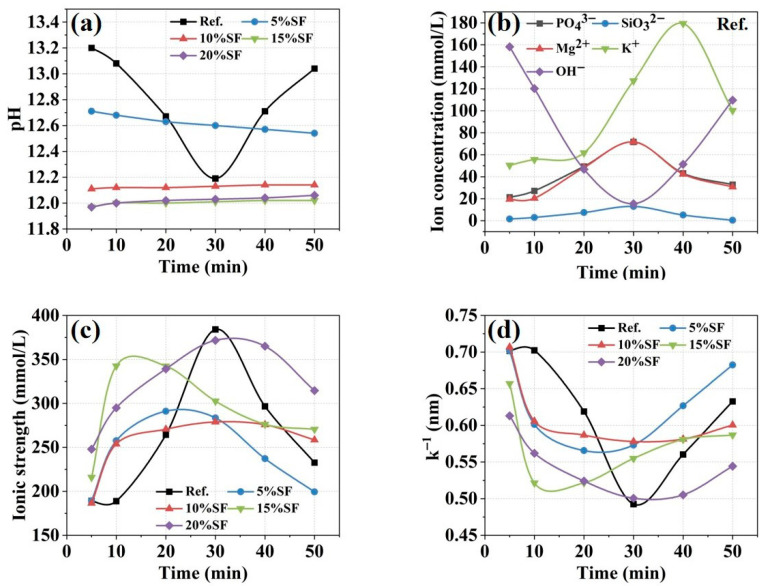
The evolution of (**a**) pH value, (**b**) ion concentration (the control group), (**c**) ionic strength, and (**d**) Debye length (k^−1^) of 3D printed MgO-SiO_2_-K_2_HPO_4_.

The pH evolution of MgO-SiO_2_-K_2_HPO_4_ system pastes as a function of hydration age and SF content is shown in Figure 8a. The control group exhibited a nonmonotonic pH change, starting at 13.20, declining to the minimum of 12.19 at 30 min, and recovering to 13.04 by 50 min, reflecting hydration dependence. This trend arose from a sequential process: the initial dissolution of MgO elevated the pH by releasing OH^−^ ions, followed by OH^−^ depletion due to K-struvite precipitation by Mg^2+^-PO_4_^3−^ binding interactions, ultimately culminating in late-stage pH stabilization by residual alkaline phase dissolution. SF incorporation generally reduced the pH of pore solution, attributed to SF-mediated acceleration of K-struvite nucleation and enhanced OH^−^ consumption efficiency [11,41].

Figure 8b shows the ion concentration evolution of pore solution in the control group, focusing on five dominant ionic species including PO_4_^3−^, Mg^2+^, K^+^, SiO_3_^2−^ and OH^−^. The concentrations of PO_4_^3−^, Mg^2+^ and K^+^ increased rapidly during early hydration, reaching a plateau of 71.69 mmol/L at 30 min, 71.70 mmol/L at 30 min and 179.39 mmol/L at 40 min, respectively, while SiO_3_^2−^ remained relatively stable between 0.42 mmol/L and 12.96 mmol/L. These trends are correlated with the dissolution of MgO and K_2_HPO_4_·3H_2_O and the precipitation of the hydration product [42].

Figure 8c,d demonstrate the variations in ionic strength and Debye length of pore solution with hydration age. The control group’s ionic strength displayed an initial increment from 189.16 mmol/L to 384.06 mmol/L within 30 min followed by gradual decline, with the peak occurring 10–20 min earlier in SF-modified systems. The reason is that SF hydrolysis generated more active sites and enhanced MgO and K_2_HPO_4_·3H_2_O dissolution through coordination effects, releasing abundant ions into the solution until soluble ions gradually formed hydration products [36]. The Debye length, which was inversely proportional to ionic strength, in the control group decreased from 0.70 nm to 0.49 nm within 30 min, followed by gradual increase. SF-induced ionic strength amplification compressed the electric double layer and reduced the Debye length, diminishing the F_EL_ and promoting the colloidal forces (F), thereby accelerating the formation of the flocculated network.

### 2.6. Discussion

Section 2.4 and Section 2.5 elucidated the evolution patterns of key parameters, including particle physical properties and electrodynamic parameters, providing critical insights into the origins of static yield stress evolution of the MgO-SiO_2_-K_2_HPO_4_ system. To elucidate the underlying mechanism, the colloidal forces and the volume fraction of K-struvite were quantified by combining experimental data with the model. A representative part of the dataset is presented, focusing on SF effects to ensure graphical clarity while retaining scientific rigor.

**Figure 9:**
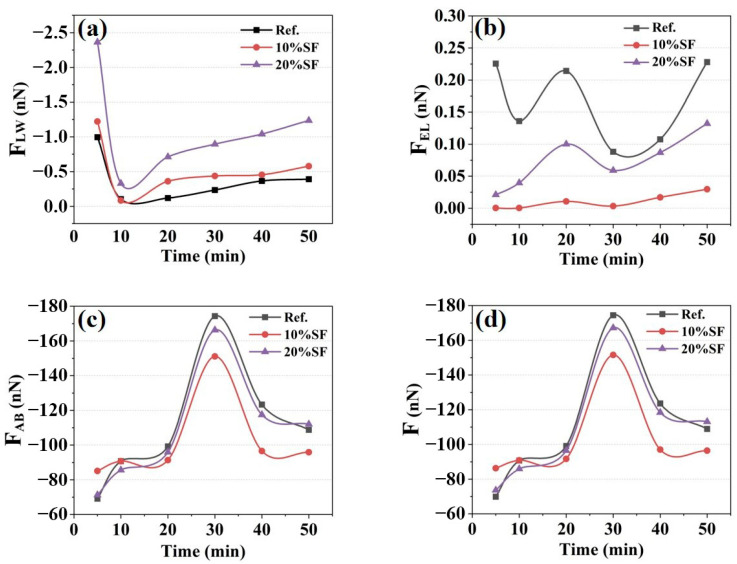
The (**a**) van der Waals force (F_LW_), (**b**) electrical double layer force (F_EL_), (**c**) acid–base interaction force (F_AB_) and (**d**) colloidal force (F) of 3D printed MgO-SiO_2_-K_2_HPO_4_.

According to the EDLVO theory, the colloidal forces (F) comprise van der Waals forces (F_LW_), electrical double layer forces (F_EL_) and acid–base interaction forces (F_AB_). A prior study [22] identified that the dominant flocculation range at particle separation was 2 nm, and F influenced the evolution of static yield stress. The values of F, F_LW_, F_EL_ and F_AB_ were calculated to reveal the relationship between static yield stress and F, as illustrated in Figure 9. The positive and negative characteristics of the values only characterized the attractive or repulsive states of the interaction between particles, while the magnitude of the absolute value reflected the intensity of the interaction.

The F_LW_ of the MgO-SiO_2_-K_2_HPO_4_ system, a dominant driver of colloidal aggregation and flow resistance during hydration, exhibited initial decrease followed by gradual increase. For the control group, the absolute value of F_LW_ was decreased from 0.99 nN at 5 min to a minimum of 0.08 nN at 10 min then rebounded to 0.39 nN at 50 min. It can be seen that the F_LW_ is mainly related to D and γ^LW^. Early hydration before 10 min triggered rapid particle interaction. Despite rapid increases in γ^LW^ and D, the difference between hydrated particles γ^LW^ and the pore solution gradually diminished. This led to a decrease in F_LW_ during this stage, reflecting a progressive convergence of particle–solution properties [43]. The F_LW_ increased steadily by incremental γ^LW^ and D adjustment between 10 and 50 min. The mixtures incorporated with SF exhibited higher F_LW_, attributable to silanol groups (Si-OH), which formed structured hydration layers that elevated γ and increased D. It was generally believed that particles with larger specific surface areas typically exhibited higher γ^LW^ [44]. The incorporation of SF reduced the specific surface area of the colloidal particles, decreasing the γ^LW^, leading to a decrease in the difference between colloidal particles and pore solution. This highlighted the capacity of SF addition to tailor interfacial energy for controlled rheological evolution.

Colloidal particles exhibit surface charge-induced electrostatic repulsion, which stabilizes suspensions by counterbalancing interparticle attraction. The F_EL_ is mainly related to D, ξ and κ^−1^. The F_EL_ displayed significant temporal fluctuations with hydration age in control mixture. For mixture with 20% SF content, the F_EL_ evolved nonmonotonically, initiating at 0.02 nN at 5 min, peaking transiently at 0.10 nN at 20 min, then rising to 0.23 nN at 50 min. Generally, the incorporation of SF reduced the F_EL_ by 19.18–99.82%, which was attributed to compressing κ^−1^ by the enhanced Mg^2+^ release and increased ξ absolute value by the generation of negatively charged M-S-H gel.

Lewis acid–base interactions, arising from electron donor–acceptor exchanges, depend on γ^AB^ and D. The absolute value of F_AB_ peaked at 30 min, with the control group measuring 174.30 nN and the 20% SF group measuring 151.11 nN, followed by a decline between 30 and 50 min, which was correlated with the higher γ^AB^ and larger D with hydration age. It can be found that the mixtures blended with 10% SF and 20% SF exhibited the lower F_AB_ primarily due to the high γ^AB^.

**Figure 10:**
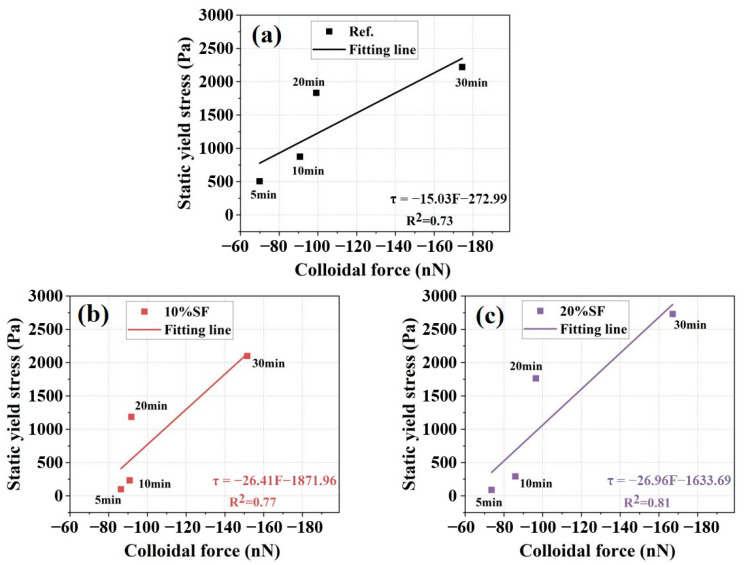
The relationship between static yield stress and colloidal force (F) of 3D printed MgO-SiO_2_-K_2_HPO_4_ with (**a**) 0% SF (**b**) 10%SF (**c**) 20% SF.

Figure 9d illustrates the evolution of F in the MgO-SiO_2_-K_2_HPO_4_ system with hydration age. The F_AB_ significantly influenced the evolution of colloidal force. Elevated ionic strength, when rapidly augmented or sustained at high concentrations, generally enhances the F. The F and static yield stress demonstrated synchronous time-dependent evolution throughout hydration progression. Particularly during Stage I (5–30 min), the rapid rise in static yield stresses was correlated with the progressive colloidal force enhancement. During Stage II (30–50 min), the decelerated growth of static yield stress partly was attributed to the reduced F. However, the experimental data showed that the effects of colloidal mechanical characteristics on rheological properties were significantly weakened, and they were no longer the decisive control parameter. The quantitative relationship between F and static yield stress in Stage I was established [45], as illustrated in Figure 10. The correlation coefficients (R^2^) were 0.73, 0.77 and 0.81 for mixtures with 0%, 10% and 20% SF, respectively. It suggested that F accounted for the evolution of static yield stress in Stage I, especially at higher SF contents. The reason for insufficient fitting performance for the current model was the inadequate consideration of early hydration.

**Figure 11:**
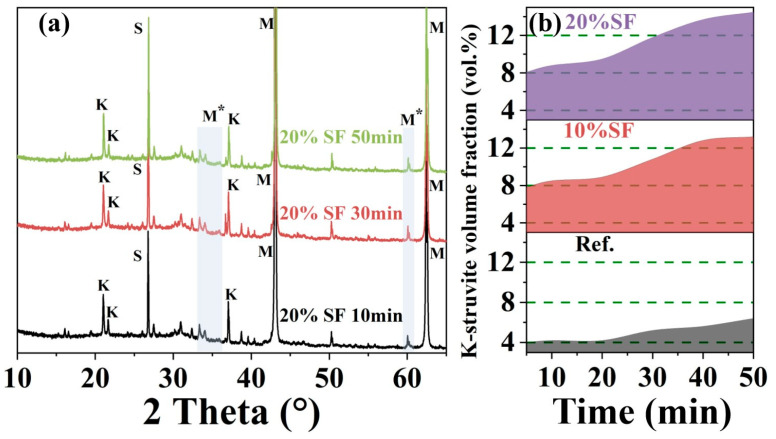
(**a**) XRD diffraction patterns of 3D printed MgO-SiO_2_-K_2_HPO_4_ with 20% SF content, (**b**) time evolution of K-struvite volume fraction of 3D printed MgO-SiO_2_-K_2_HPO_4_. M: Magnesia (MgO), M*: M-S-H gel, K: K-struvite (MgKPO_4_·6H_2_O), S: SiO_2_.

Hydration product formation exerts an effect on static yield stress evolution in the MgO-SiO_2_-K_2_HPO_4_ system, exhibiting a strong correlation between the time-varying structural strength and the dynamic accumulation of volume fraction of the hydration product [20]. Quantitative XRD-Rietveld analysis revealed the time evolution of volume fraction of K-struvite (primary hydration product), as illustrated in Figure 11. Initial hydration within 5 min produced K-struvite instantaneously, followed by a growth rate which surged at 20 min, which was associated with the fitting deviation of the static yield stress–F coupling model at 20 min. When SF contents increased from 0% to 20%, the volume fraction of K-struvite increased. The mechanism can be related to the hydration reaction path.

Wang et al. [42] proposed three distinct pathways for the formation of K-struvite in MKPCs using PDP as the acidic component. Pathway I: Rapid dissolution of MgO and PDP released abundant soluble ions, enabling direct precipitation of K-struvite. Pathway II: Newberyite (MgHPO_4_·3H_2_O) transiently crystallized, followed by its conversion to K-struvite under pH > 7 and favorable ionic concentration conditions. Pathway III: The potassium-deficient phosphate amorphous crystallized to form K-struvite. In this study, K_2_HPO_4_·3H_2_O rather than PDP was employed to prepare MKPCs. Although the initial precipitation kinetic of K-struvite in the system may be similar to the above pathways, the early nucleation mechanism has not been fully elucidated.

In 3D printed MgO-SiO_2_-K_2_HPO_4_ in this study, the dominant reaction path of hydration products is preceded by soluble ion coordination (Equations (2)–(4)) [11,23], governed by a heterogeneous nucleation mechanism. MgO is reacted with water to form surface-bound [Mg(H_2_O)_6_^2+^] complexes initially, leading to the precipitation of K-struvite through K^+^ and PO_4_^3−^ ions binding at interfacial sites [46].

Notably, the conversion pathway of Newberyite to K-struvite was absent. The supersaturation ratio β(Newberyite)/β(K-struvite) of the solution dictated which substance precipitated from the solution, and it was strongly influenced by the pore solution pH [47]. The pH quickly jumped above 7 in the initial phase, which exceeded the critical pH threshold and prevented the nucleation of Newberyite. In addition, the high concentration HPO_4_^2−^ resulting from the dissociation (Equation (1)) differed from the H_2_PO_4_^−^ required to form the Newberyite. According to the reaction equation, the M/P ratio of 1.0 was sufficient for the formation of K-struvite. The high Mg/P ratio (8:1) inhibited the nucleation of potassium-free phosphate hydrates such as MgHPO_4_·3H_2_O by reducing the concentration of phosphate [48]. The low water–cement ratio (W/C = 0.5) further amplified the concentration of Mg^2+^ and K^+^ ions, accelerating the reaction kinetics. Optimization to M/P = 3.0 and W/C = 0.14 further amplified the selectivity of K-struvite, aligning with the XRD results that proved that the Newberyite did not exist. The result was consistent with the theoretical prediction.

**Figure 12:**
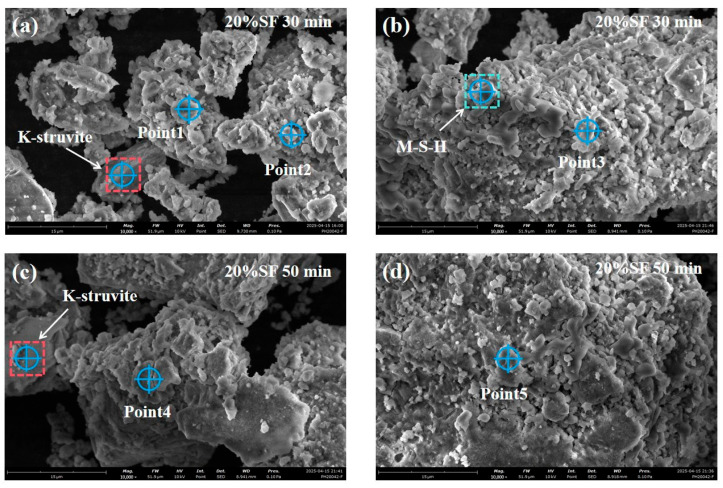
(**a**–**d**) SEM images of 3D printed MgO-SiO_2_-K_2_HPO_4_ with 20% SF at 30 and 50 min.

The last pathway involved the crystallization of potassium-deficient phosphate amorphous phase, which was undetectable by XRD. SEM-EDS analysis was conducted on 20% SF mixtures at hydration ages of 30 and 50 min. Figure 12 reveals that irregular fine particles were adhered to particle surfaces and identified as amorphous phases. Table 1 shows that the elemental quantification included O, Mg, P and K, at points of 1–5. The obtained atom counts fluctuated but were acceptable. They were confirmed to be amorphous phases rich in O and Mg but poor in K and P, and the average molar ratio of elements was consistent with the transition of potassium-deficient phosphate mineral [42]. In addition, concurrent formation of crystalline K-struvite and M-S-H gel (Mg/Si ≈ 0.67–1.00) were also confirmed in Figure 12.

MgO + H_2_PO_4_^−^ + 3H_2_O → MgHPO_4_·3H_2_O + OH^−^ (1)

Mg(OH)_2_ → Mg^2+^ + 2OH^−^ (2)

K_2_HPO_4_·3H_2_O → 2K^+^ + HPO_4_^2−^ + 3H_2_O (3)

K^+^ + PO_4_^3−^ + Mg^2+^ + 6H_2_O → MgKPO_4_·6H_2_O (4)

Revisiting Figure 11b, K-struvite formation between 5 and 50 min followed the multiphasic crystallization mechanism, combining direct ion precipitation and amorphous-to-crystalline conversion from potassium-deficient phosphate mineral [42]. Phase quantification revealed that the relative volume fraction of K-struvite maintained relatively stable or slow increase during Stage I (5–30 min) and the correlation between the volume fraction of K-struvite and static yield stress was low, confirming the colloidal dominance. During Stage II (30–50 min), the volume fraction of K-struvite increased sharply while the characteristic parameters of colloidal force decreased, indicating that the hydrates network was the dominant cause of static yield stress.

The incorporation of SF regulated the crystallization path of K-struvite through dual dynamic effects. Firstly, SF accelerated the dissolution of MgO, leading to an increase in the concentration of Mg^2+^ in solution and amplifying ion-driven nucleation. Secondly, the higher the SF content, the faster the SiO_2_ dissociation rate. More amorphous phase was generated and then transformed into more K-struvite, promoting the non-classical crystallization pathway. Furthermore, the mixture with 20% SF achieved the increased static yield stress during Stage II through microstructural densification, with the interlocking M-S-H gel and larger layered K-struvite filling the voids left by unreacted raw materials [49].

**Figure 13:**
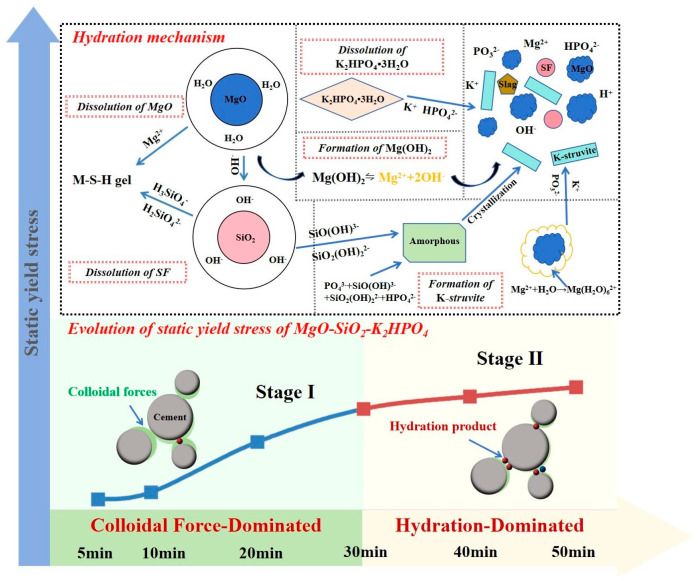
Schematic of static yield stress evolution of 3D printed MgO-SiO_2_-K_2_HPO_4_.

The evolution of static yield stress in MgO-SiO_2_-K_2_HPO_4_ containing 20% SF can be categorized into two distinct stages, illustrating concurrent M-S-H gel formation alongside K-struvite crystallization, as illustrated in Figure 13. Stage I (0–30 min): The evolution of static yield stress was predominantly governed by the colloidal forces generated between cement particles. Stage II (30–50 min): The formation of the structural network with K-struvite as the main hydration product became the decisive factor for the static yield stress.

A critical consideration in this study is the focus on theoretical K-struvite volume fraction for hydration mechanism interpretation. Although the K-struvite volume fraction-based model offered initial insights, the utility was constrained by some limitations. Firstly, omitting M-S-H gel and slag-derived C-A-S-H gel exerted influences on structural network development through competitive reaction. Secondly, some inaccuracy had been reported in the contribution of volume fraction of hydration products [50]. Zhang et al. [20] established quantitative relationships between C-S-H particle interaction forces and pastes microstructure in Portland cement systems, a methodological framework that remains to be systematically extended to the MgO-SiO_2_-K_2_HPO_4_ system. Future investigations should prioritize nanomechanical characterization of interparticle forces among hydration products including K-struvite, M-S-H and C-A-S-H gel, establishing direct linkages between the microscopic interactions and the macroscopic static yield stress evolution observed in Stage II through network formation.

Pore solution analysis confronted the methodological limitations. The ICP measurements equated total elemental P with the concentration of PO_4_^3−^, which was imprecise to the MgO-SiO_2_-K_2_HPO_4_ system’s hydration chemistry. This assumption neglected phosphorus speciation (e.g., HPO_4_^2−^, H_2_PO_4_^−^) and dynamic ion equilibria, and thus led to analytical inaccuracy. Furthermore, variable ionic valence states perturbed electric double layer force calculations, affecting colloidal interaction models. Also, electric double layer forces play a key role in colloidal forces; the precise quantification is vital for understanding the retardation mechanism. Future studies should combine advanced techniques with EDLVO theory to study ionic valence impacts on hydration kinetic and microstructural evolution.

## 3. Conclusions

The hydration characteristics, rheological behavior and mechanical property of 3D printed MKPCs of MgO-SiO_2_-K_2_HPO_4_ were systematically studied. This study elucidates the colloidal–crystallization synergy mechanism of silica fume (SF) governing rheological evolution. Four main results can be summarized as follows:

(1) The MgO-SiO_2_-K_2_HPO_4_ system with 20% SF achieved synergistic rheo-mechanical balance without triggering excessively rapid setting time, resolving the trade-off between early-stage rheological properties and long-term strength, demonstrating superior applicability for 3D printing.
(2) The evolution of static yield stress within 50 min consisted of two stages, which corresponded to rapid linear growth in 0–30 min (Stage I) and slow increased plateau in 30–50 min (Stage II). Colloidal forces driven by EDLVO interactions dominated Stage I, while hydration network strength controlled Stage II by K-struvite crystallization.
(3) The main driving force of increased static yield stress in Stage II was related to crystallization pathways of K-struvite, including direct precipitation from soluble Mg^2+^, K^+^, PO_4_^3−^ coordination and non-classical crystallization of transformation from the potassium-deficient phosphate amorphous phase.
(4) The incorporation of SF influenced static yield stress through enhancing the colloidal forces by M-S-H gel formation which altered interparticle forces and system stability, and increased hydration network strength by accelerating hydration.

The quantitative analysis of the effects of colloidal forces and hydration kinetic governing static yield stress evolution provides an understanding of the mechanism for early microstructural construction. This establishes a predictive framework for structural stability control and rheologically improved 3D printed MKPCs.

## 4. Materials and Methods

### 4.1. Raw Materials and Sample Preparation

**Figure 14:**
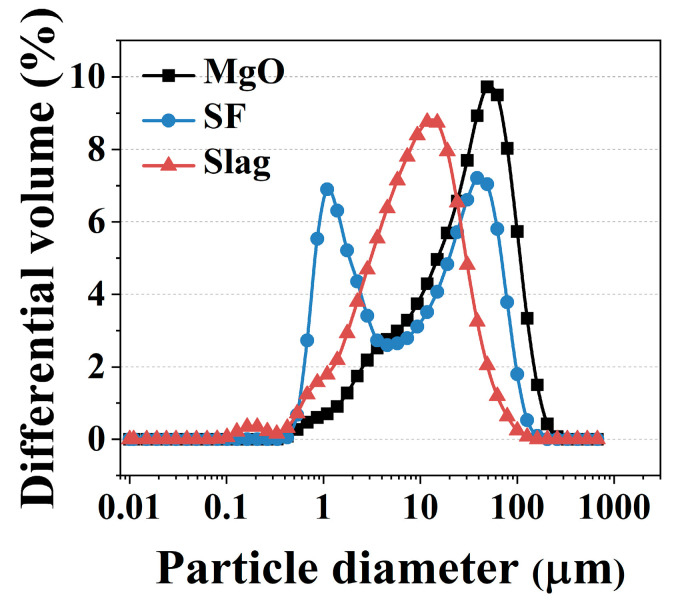
Particle size distribution of raw material.

The raw materials are dead burnt MgO, K_2_HPO_4_·3H_2_O, SF and slag powders. The chemical compositions characterized by XRF analysis are given in Table 2 and the particle size distribution is shown in Figure 14.

Table 3 details the formulations of the 3D-printable MgO-SiO_2_-K_2_HPO_4_ system. The mixtures are prepared by a three-stage mixing protocol: (1) high-speed mixing (120 rpm, 60 s) of dead burnt MgO, K_2_HPO_4_·3H_2_O, SF and water; (2) addition of quartz sand and slag powders under low-speed mixing (60 rpm, 30 s).; and (3) final high-speed mixing (120 rpm, 150 s) to ensure uniform dispersion.

### 4.2. Rheological Property Tests

**Figure 15:**
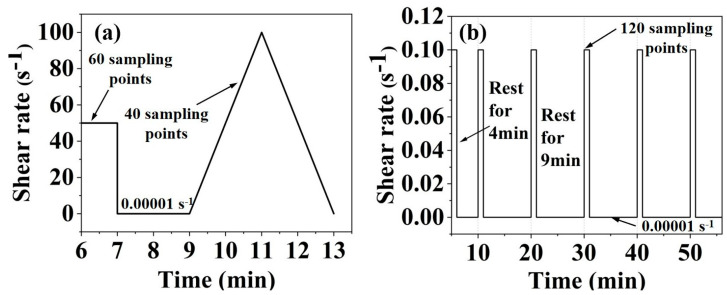
Schematic plots of (**a**) dynamic shear test protocol and (**b**) static shear protocol.

The rheometer (HAAKE MARS 40) was employed in this study. As shown in Figure 15a, a shear rate control method was employed to evaluate the dynamic yield properties of the slurry. The testing procedure consisted of pre-sheared at a constant shear rate of 50 s^−1^ for 60 s and rested for 120 s, and then the variable shear rate of 0.00001–100 s^−1^ (100–0.00001 s^−1^) for 240 s. The Bingham model was employed in this study. The intercept of the fitted line corresponds to the dynamic yield stress while the slope represents the plastic viscosity.

The variable shear rate was from 0.00001 to 100 s^−1^ and the thixotropy of the slurry was characterized by the area of the hysteresis loop enclosed by the upward and downward shear stress curves. The structural parameter was quantified from the ratio of peak shear stress (τ_1_) to steady-state shear stress (τ_2_) during the initial loading phase of dynamic rheological testing, conducted at a constant shear rate of 50 s^−1^.

Monitoring the time evolution of the static yield stress of the MgO-SiO_2_-K_2_HPO_4_ system is the key indicator for assessing the buildability. As shown in Figure 15b, the static shear protocol involves the application of quasi-static shear conditions (0.1 s^−1^ for 60 s) at predefined intervals (5, 10, 20, 30, 40, 50 min). The peak shear stress during each interval corresponds to the static yield stress.

### 4.3. Setting Time Tests

The initial and final setting times of fresh slurry were determined using Vicat apparatus, with respective setting points defined at needle penetration depths of 36 ± 1 mm and 40 ± 1 mm.

### 4.4. Sample Preparation

The solvent displacement method was employed to stop the hydration process. At each timepoint, 3 g of fresh paste was immersed in anhydrous isopropanol under mechanical agitation (500 rpm, 15 min) to displace unbound water and terminate cementitious reactions. The resultant suspension was subjected to vacuum filtration and rinsed again with isopropanol solution. Finally, the obtained wet powders were placed in a vacuum drying oven and dried at 35 °C for 12 h.

### 4.5. Particle Properties Tests

Particle size distribution and specific surface area were quantified by laser diffraction analysis using anhydrous ethanol as a non-polar dispersant to minimize interparticle agglomeration during sonication [20,28,45].

The temporal surface energy of hydrated powders was quantified through thin-layer wicking analysis predicated on the Washburn equation [51,52]. This approach exploits capillary penetration dynamics, where a test liquid advances through a uniform porous thin layer (deposited on a glass substrate) at a rate governed by the interfacial energetics of the material.

The procedure consists of: (1) A very low surface energy solution (commonly n-heptane, regarded as cosθ = 1) was utilized to permeate the plate containing dry powders at a specific hydration age to obtain t-x^2^ curves, which were integrated with Equation (5) to calculate the Reff of the employed plate. Critical to this protocol, the glass plate was adequately pre-contacted with the vapors of the solution prior to permeation to form a double film, thereby ensuring that θ = 0. (2) Polar solutions (formamide, water and bromonaphthalene) were employed to permeate the plate and the contact angles of various polar solutions on the powder were calculated based on the Reff and the derived t-x^2^ curves. Further, the dynamic forward and dynamic backward angles were determined using untreated and vapor-preconditioned plates; the average value was considered as the contact angle. (3) The surface energy of the powder was determined using Equations (6) and (7). Finally, the contact angles of three polar probe liquids (water, formamide and bromonaphthalene), along with the relevant parameters of the polar solutions, can be derived from the ternary equation featuring three unknowns, ascertaining the surface energy of the powders and the associated values [53].

(5) $$x^{2}=\frac{R_{\mathrm{eff}}t}{2\eta_{l}}\gamma_{l}\cos\theta$$

where x is liquid front penetration distance; t refers to the penetration time; η_l_ is the viscosity of the test liquid; γ_l_ represents the surface tension of the test liquid; and θ refers to the contact angle.

(6) $$\gamma =\gamma^{\mathrm{LW}}+\;\gamma^{\mathrm{AB}}=\gamma^{\mathrm{LW}}+2\sqrt{\gamma^{+}\gamma^{-}}$$

(7) $$\gamma_{l}\left(1+\cos\theta \right)=2\left(\sqrt{{\gamma_{s}}^{\mathrm{LW}}{\gamma_{l}}^{\mathrm{LW}}}+\sqrt{{\gamma_{s}}^{+}{\gamma_{l}}^{-}}+\sqrt{{\gamma_{s}}^{-}{\gamma_{l}}^{+}}\right)$$

where γ is the surface energy (mJ/m^2^); γ^LW^ is the apolar parameter of surface energy (mJ/m^2^); γ^AB^ is the polar parameter of surface energy (mJ/m^2^); γ^+^ is the Lewis acid parameter of γ^AB^ (mJ/m^2^); γ^−^ is the Lewis base parameter of γ^AB^ (mJ/m^2^); and s and l represent the solid phase and liquid phase, respectively.

### 4.6. Electrodynamic Parameter Tests

The zeta potential of the pastes was measured by an electroacoustic technique (ZetaProbe Analyzer, Malvern Instruments LTD, Malvern City, UK). Specimens at targeted hydration ages were dispersed in anhydrous ethanol and transferred into the tube prior to measurement.

For pore solution analysis, pastes specimens were separated into the liquid phase through centrifugation (300 g, 5 min), which was chemically stabilized through acidification (pH = 1.0) for ICP-OES. Hydroxide ion concentration ([OH^−^]) was calculated indirectly from pH.

The ionic strength of the pore solution and the corresponding Debye length were calculated using Equations (8) and (9), respectively.

(8) $$I=\frac{\sum c_{i}z_{i}^{2}}{2}\;\left(\mathrm{mol}/L\right)$$

where I refers to the ionic strength, c_i_ refers to the concentration of each ion and z_i_ refers to the charge number of the ion.

(9) $$\kappa^{-1}=\sqrt{\frac{\varepsilon_{0}\varepsilon_{r}K_{B}T}{e^{2}\sum_{i}{n_{i}z}_{i}^{2}}}$$

where κ^−1^ refers to the Debye length (m); ε_0_ refers to the vacuum permittivity (8.854 × 10^−12^ C^2^·J^−1^·m^−1^); ε_r_ refers to the relative permittivity of water (80.18, 20 °C); K_B_ refers to Boltzmann’s constant (1.38 × 10^−23^ J/K); T refers to the absolute temperature (293.15 K, 20 °C); e refers to the elementary charge (1.6 × 10^−19^ C); and n_i_ refers to the number density of the ion.

### 4.7. Microstructure Characterization

Phase composition was characterized by XRD with Cu Kα radiation. Diffractograms were acquired over an angular range of 5–70° 2θ, with a step size of 0.024°. Rietveld refinement was employed for quantitative phase analysis. Fresh fracture surfaces were imaged using scanning electron microscopy (SEM, Pharson XL, Phenom-World Holding B.V, Eindhoven, The Netherlands.) at 5 kV acceleration voltage. Mortar specimens (40 × 40 × 40 mm^3^) were cast, cured at 20 °C/70% RH for 24 h, then demolded and stored in 20 °C and 95% RH. Compressive strength tests were conducted at 1, 3, 7 and 28 d by a universal testing machine (CDT1305-2, MTS Systems Corporation, Eden Prairie, MN, USA) at a loading rate of 0.5 MPa/s.

### 4.8. Interparticle Forces Calculation

Assuming the particles are spherical, the van der Waals forces (F_LW_) between the two particles can be calculated as follows [22]:

(10) $$F_{\mathrm{LW}}\;=\;\frac{A}{12h^{2}}\frac{D_{1}D_{2}}{D_{1}\;+\;D_{2}}$$

(11) $$A=\;-12\pi {h_{0}}^{2}\Delta G_{h_{0}}^{\mathrm{LW}}$$

(12) $$\Delta G_{h_{0}}^{\mathrm{LW}}=2\left(\sqrt{\gamma_{s_{1}}^{\mathrm{LW}}}\;-\;\sqrt{\gamma_{1}^{\mathrm{LW}}}\right)\left(\sqrt{\gamma_{s_{2}}^{\mathrm{LW}}}\;-\;\sqrt{\gamma_{1}^{\mathrm{LW}}}\right)$$

where A represents the Hamaker constant for interacting media, J; h represents the particle-to-particle separation distance, m; $D_{1}$, $D_{2}$ are the diameters of interacting particles, m; $h_{0}$ represents the minimum separation distance caused by the Born repulsion (0.158 nm); and $\Delta G_{h0}^{\mathrm{LW}}$ represents the Lifshitzvan der Waals free energy density, mJ/m^2^.

The electrical double layer forces (F_EL_) can be calculated as follows [54]:

(13) $$F_{\mathrm{EL}}\;=\;\frac{D_{1}D_{2}}{D_{1\;}+\;D_{2}}\frac{\pi \varepsilon_{0}\varepsilon_{r}\kappa \exp\left(-\kappa h\right)}{1\;-\;\exp\left(-2\kappa h\right)}\left\{2\xi_{1}\xi_{2}-\;\left(\xi_{1}^{2}\;+{\;\xi }_{2}^{2}\right)\exp\left(-\kappa h\right)\right\}$$

where $\xi_{1}$ and $\xi_{2}$ are the surface potential approximated by the experimentally measured zeta potential, v; and κ represents the reciprocal of Debye length, m^−1^.

The acid–base interaction forces (F_AB_) can be calculated as follows [20]:

(14) $$F_{\mathrm{AB}}\;=\;\pi \frac{D_{1}D_{2}}{D_{1}\;+\;D_{2}}\Delta G_{h_{0}}^{\mathrm{AB}}\exp\left(-\frac{h}{\lambda_{\mathrm{AB}}}\right)$$

(15) $$\Delta G_{h_{0}}^{\mathrm{AB}}=2\sqrt{\gamma_{l}^{+}}\left(\sqrt{\gamma_{s1}^{-}\;}+\sqrt{\gamma_{s2}^{-}}\;-\;\sqrt{\gamma_{l}^{-}}\right)+2\sqrt{\gamma_{l}^{-}}\left(\sqrt{\gamma_{s1}^{+}\;}+\sqrt{\gamma_{s2}^{+}}\;-\;\sqrt{\gamma_{l}^{+}}\right)\;-\;2(\sqrt{\gamma_{s1}^{+}}\sqrt{\gamma_{s2\;}^{-}}+\sqrt{\gamma_{s1}^{-}}\sqrt{\gamma_{s2}^{+}})$$

where $\Delta G_{h_{0}}^{\mathrm{AB}}$ is the acid–base adhesion energy density, mJ/m^2^; and λ_AB_ is the molecule correlation length in water (0.6 nm).

The colloidal forces (F) can be calculated as follows:

F = F_LW_ + F_EL_ + F_AB_ (16)

## Figures and Tables

**Table 1:** EDS semi-qualitative results for distinct zones.

EDS Zone	Atom Counts (%)			
	O	Mg	P	K
Point1	34.49	47.20	3.39	9.74
Point2	53.62	40.48	2.04	2.61
Point3	52.11	28.83	2.99	12.27
Point4	54.51	37.29	1.38	2.93
Point5	62.94	23.68	4.16	7.39
Average	51.53	35.50	2.79	6.99

**Table 2:** Chemical compositions of magnesia, SF and slag (wt.%).

Material	MgO	CaO	SiO_2_	Al_2_O_3_	P_2_O_5_	Fe_2_O_3_	K_2_O	SO_3_	Others
Magnesia	90.27	0.95	3.47	0.60	0.23	1.74	0.10	0.09	2.55
SF	0.71	0.02	96.72	1.33	0.63	0.01	0.09	0.03	0.46
Slag	10.68	41.52	24.99	17.28	0.09	0.73	0.38	1.57	2.76

**Table 3:** Mix proportions of 3D printed MKPCs (wt.%).

	MgO	K_2_HPO_4_·3H_2_O	SF	Slag	W/B	S/B
Ref.	67.5	22.5	0	10	0.14	0.4
5%SF	63.75	21.25	5	10		
10%SF	60	20	10	10		
15%SF	56.25	18.75	15	10		
20%SF	52.5	17.5	20	10		
25%SF	48.75	16.25	25	10		

Note: Magnesium/phosphate (M/P) mass ratio is 3.0, water/binder (W/B) ratio is 0.14 and sand/binder ratio (S/B) is 0.40.

## Data Availability

The original contributions presented in this study are included in the article. Further inquiries can be directed to the corresponding author.
